# Concentration of traffic air pollutants and influencing metrological factors in Hawassa City roadways, Ethiopia

**DOI:** 10.3389/fpubh.2024.1510194

**Published:** 2025-01-29

**Authors:** Asmare Asrat Yirdaw, Amanuel Ejeso, Samrawit Mokie Belayneh, Lamrot Yohannes, Anmut Endalkachew Bezie, Embialle Mengistie Beyene

**Affiliations:** ^1^Departments of Environmental Health, School of Public Health, College of Medicine and Health Science, Arba Minch University, Arba Minch, Ethiopia; ^2^Department of Environmental Health, College of Medicine and Health Science, Hawassa University, Hawassa, Ethiopia; ^3^Department of Environmental and Occupational Health and Safety, Institute of Public Health, College of Medicine and Health Science, University of Gondar, Gondar, Ethiopia; ^4^Department of Occupational Health and Safety, College of Medicine and Health Sciences, Wollo University, Dessie, Ethiopia

**Keywords:** transport, particulate matter, nitrogen dioxide, traffic air, pollutants

## Abstract

**Introduction:**

The traffic air pollution caused by transportation is a growing global problem that contributes to millions of deaths each year. Despite its importance, information on pollutant concentration is limited in many developing cities, especially in Ethiopia. This study aimed to determine the concentration levels and spatial and temporal variations of traffic air pollutants in Hawassa and to investigate the influence of metrological parameters on the concentration of traffic air pollutants.

**Methods:**

A real-time monitoring system of Aero-Qual Series 300/500 was used to monitor pollutants, and 24 monitoring sites were included on both heavy and low-traffic volume roads. The study monitored morning and afternoon times over 24 days to comprehensively characterize the temporal variations.

**Results:**

The results showed that the mean PM_2.5_ concentration on heavy- and low-traffic volume roads was 161.6 ± 26.1 μg/m^3^ and 95 ± 14.2 μg/m^3^, respectively, whereas the PM_10_ concentration was 178.7 ± 20.3 μg/ m^3^ and 102.3 ± 17.6 μg/m^3^, respectively. Similarly, the mean NO_2_ concentrations on roads with heavy and low traffic volumes were 86.4 ± 14.4 μg/m^3^ and 61.7 ± 14.2 μg/m^3^, respectively. Significantly higher, concentrations were recorded on traffic light roads, followed by main asphalt roads, for both types of traffic air pollutants. The ratio of PM_2.5_/PM_10_ was higher (0.924), in which the pollution sources attributed to anthropogenic sources. Kendall’s tau-b correlation analysis suggested that Meteorological parameters (temperature and relative humidity) were positively correlated with traffic air pollutants. Likewise, stepwise multiple linear regression analysis confirms that the concentrations of traffic air pollutants had a positive relationship with metrological parameters.

**Implications:**

The findings of this study therefore showed the need for regular air quality monitoring of the urban areas to copping out the adverse public health impacts. And, it highlighted an urgent need for long-term monitoring of traffic air pollution and the development of emission control programs that can be readily implemented to decrease the emissions from anthropogenic sources. Also, it brings a sense of collaboration among stakeholders to tackle the effects of air pollution by providing an inclusive and sustainable development agenda for Hawassa.

## Introduction

Ambient air pollution causes 4.2 million premature deaths worldwide every year, of which 91% occur in low- and middle-income countries (LMICs). Notably, 20% of these deaths are due to air pollution from road traffic (1, 2). The number of deaths caused by air pollutants, particularly particulate matter (PM_2.5_), exceeds 4.2 million per year and accounts for 7.6% of global deaths (3). Traffic air pollution is a pressing global problem, especially in LMICs (1) and exposure to ambient PM is a major public health concern (4). Air pollution is considered one of the greatest threats to public health worldwide, and the health problems commonly associated with air pollution are chronic diseases (5, 6).

Ambient air pollution causes a range of minor upper respiratory irritations to serious chronic respiratory and cardiac diseases (7), from aggravation of pre-existing heart and lung problems to premature mortality and, reduced life expectancy. These adverse health effects are associated with exposure to PM, NO_2_ and long-term high-concentration exposure to PM leads to an increased risk of lung cancer, respiratory disease, and arteriosclerosis, whereas short-term exposure to PM can cause exacerbation of several forms of respiratory diseases and changes in heart rate variability (8). As a consequence, ambient air pollution, especially PM exposure, is more severe than ever (9, 10). Sub-Saharan African (SSA) countries are undergoing an epidemiological transition, manifested by a substantial burden of both communicable and non-communicable diseases (NCDs). The increase in NCDs is associated with the risk factors that accompany lifestyle changes and the expansion of urbanization (7, 10, 11).

Six criteria pollutants, namely particulate matter (PM), carbon monoxide (CO), sulfur dioxide (SO_2_), nitrogen dioxide (NO_2_), lead, and ozone (O_3_), have been identified as major public health concerns (6). Road traffic contributes significantly to air pollution and is responsible for 7.7%, 10%, and 28% of PM_10_, PM_2.5_, and NO_x_ emissions, respectively (12). In particular, emissions from motor vehicles are the main source of NO_2_, an indicator of traffic air pollution in urban areas (13). Road traffic is also the major source and contributor of black carbon and PM_2.5_ (88%) in four West African cities (14). In particular, road traffic is a major source of urban PM and atmospheric metals, and air quality experts have recently focused on this sector for specific emission control measures (8). The PM released from road traffic in Sub-Saharan African (SSA) countries is higher compared to developed countries (7). For example, the PM_2.5_ concentration in the United States was 9 μg/m^3^ in 2019, and the concentration level in seven African countries ranged from 40 to 260 μg/m^3^ (11). Most African countries predominantly use second-hand vehicles and poorly maintained old cars, and the frequent stop-and-go of the vehicles contributes to the emissions of traffic air pollutants like NO_2_ and PM (15). There is also a significant usage of two-wheel vehicles for public transportation, and a lack of urban planning causes severe traffic congestion, which ultimately causes an increase in traffic air pollution in urban settings (9–11).

Data on traffic air pollutants are limited, especially in low-and middle-income countries. Although most African studies have found exceedances of the World Health Organization (WHO) limits on particulate matter (PM) and nitrogen dioxide (NO_2_) (5), the available data are limited and scattered. However, recent research has provided some insight into the scale of PM_2.5_ contamination. PM_2.5_ concentrations in low-income countries (LICs), low-and middle-income countries (LMICs), and high-income countries (HICs) were 78 μg/m^3^, 55 μg/m^3^, and 14 μg/m^3^, respectively (1, 16). In addition, data from 2019 showed that around 80% of the urban population lived in areas where the WHO limits for ambient air pollutants were exceeded, while data from 2018 showed that 93% of urban children lived in areas where the WHO limits were exceeded (1, 17). Previous studies have shown that PM and NO_2_ concentrations varied across heavy- and low-traffic exposure roads. For example, studies conducted in the USA (18), Norway (19), Uganda (5), and a local study in Addis Ababa, Ethiopia (20) showed that the concentrations of those pollutants greatly varied across road types. Studies conducted in Malaysia (21), Nigeria (22), and Ethiopia (23) have shown that concentrations of air pollutants were higher in the morning than in the afternoon.

Although industrially developed countries have made a continuous effort to reduce exposure to air pollution, mortality and morbidity associated with air pollution have not decreased on a global level (9). To tackle the effects of traffic air pollution, restricting rules on vehicles and fuel usage is vital. Public transportation and infrastructure for walking and bicycling should be encouraged. Some cities in Africa are initiating stricter rules, demonstrating that local governments play a key role in mitigating air pollution. For example; South Africa’s Air Quality Act (Act 39 of 2004), allows local governments to create their standards (24, 25). The trans-boundary nature of air pollution is a problem for many African countries, and a binding rule concerning air pollution on a global level is needed (26). Likewise, the Ethiopian government has implemented various strategies to address the impact of the transportation sector on air quality, as outlined in its policy. Measures include the blending of 5% ethanol into gasoline, with plans to increase the proportion to 25% in the future (27). The government has also promoted non-motorized transport and banned the import of leaded petrol (28). Despite these efforts, the concentration of air pollution has not decreased significantly.

In Ethiopia, however, data on traffic air pollutants are limited. As far as the researcher is aware, only three publications have addressed the concentrations of PM and NO_2_, with average 30-min concentrations of PM_2.5_ and PM_10_ of 30 μg/m^3^ and 59 μg/m^3^, respectively (28–30). In addition, a recent study in Ethiopia found that PM_10_ concentrations near roads and roadsides “exceed 50% of WHO limits” (28). The aim of the current study is therefore to gain new insights into the concentration of traffic air pollutants (NO_2_, PM_2.5_, and PM_10_) in different road types and to identify potential hotspots of air pollution in the Ethiopian City of Hawassa, thus closing an important knowledge gap.

## Materials and methods

### Study area

Hawassa, the capital of the Sidama region, was the site of an air pollution monitoring study. The City is located 273 km south of Addis Ababa at latitude of 07°15′N, a longitude of 38°45′E, and an altitude of 1,708 m above sea level. The City experiences an extended rainy season from March to October with an average annual rainfall of 950 mm, with 44% of the rainfall occurring between June and September. The climate of Hawassa can be categorized as dry to sub-humid, with temperatures ranging from 9°C to 29°C, and an average temperature of 23°C, relative humidity of 60% (63).

According to the Hawassa Transport Authority, the City has a total road network of 1983 km, including 152 km of asphalt, 620 km of gravel, 511 km of dry weather roads, 240 km of red ash, and 460 km of cobblestone. The total area of all roads was 10.26 km^2^, of which 36% was asphalt, 48% was gravel (compressed earth and red ash), and the remaining 16% was covered by cobblestone.

### Study design and period

In this study, a comparative cross-sectional study design was used to determine the concentration levels and spatial and temporal variations of selected traffic-related air pollutants (NO_2_, PM_2.5_, and PM_10_) in six different road types and Influencing Metrological Parameters in Hawassa City roadways, Ethiopia, from March 20, 2023, to April 14, 2023.

### Sample size determination and sampling techniques

The sample size was determined by purposive sampling technique, whereby roads with heavy and low traffic volumes were selected. Two main sampling stations were defined: Stations for heavy-traffic volume roads, including traffic light roads and main asphalt roads, and stations for low-traffic volume roads. Generally, we included five traffic light roads, seven main asphalt roads, and twelve low-traffic volume roads (gravel, cobblestone, dry weather, and red ash roads); three sites were included from each low-traffic volume road. This resulted in 24 monitoring sites being used for each pollutant.

The monitoring sites were purposively selected based on careful average traffic flow counts before monitoring and peak traffic hours (7:00 to 9:00 am and 4:00 to 5:30 pm) as sampling times in the case of the City of Hawassa. To comprehensively characterize the temporal variations in pollutant concentrations, sampling was conducted in two phases, with one-hour monitoring taken during each of the morning and afternoon peak hours (7:00 to 9:00 am and 4:30 to 5:30 pm). We monitor the traffic air both in the morning and afternoon for a one-hour duration at each sampling site. After measuring the air pollutant concentration at each sampling point at 3-min intervals, the mean pollutant concentrations during 15 min, 30 min, and 1-h in each study area were calculated as descriptive statistics using the general formula as follows Equations 1–3 (20).

(1) $$\begin{array}{l} \mathrm{Average concentrations}\\ =\frac{\mathrm{summation}\;\mathrm{of}\;\mathrm{pollutant}\;\mathrm{concentration}\;\mathrm{recorded}\;\mathrm{within}\;15\;\min}{5\left(\mathrm{number}\;\mathrm{of}\;\mathrm{record}\;\mathrm{within}\;15\;\min\right)\;} \end{array}$$

(2) $$\begin{array}{l} \mathrm{Average concentrations}\\ =\frac{\mathrm{Summation}\;\mathrm{of}\;\mathrm{polutants}\;\mathrm{concentration}\;\mathrm{within}\;30\;\min}{10\left(\mathrm{number}\;\mathrm{of}\;\mathrm{record}\;\mathrm{within}\;30\;\min\right)} \end{array}$$

(3) $$\begin{array}{l} \mathrm{Average concentrations}\\ =\frac{\mathrm{Summation}\;\mathrm{of}\;\mathrm{pollutants}\;\mathrm{concentration}\;\mathrm{within}\;60\;\min}{20\left(\mathrm{number}\;\mathrm{of}\;\mathrm{record}\;\mathrm{within}\;60\;\min\right)} \end{array}$$

### Operational definition

Heavy traffic flow areas were defined as areas where the average daily traffic volume was more than or equal to 18,000 vehicles (750 vehicles/h), and Low traffic flow areas refer to: areas where the average daily traffic volume was less than or equal to 2,800 vehicles (117 vehicles/h) (31).

### Data collection tools and procedures

Air quality data were monitored using Aero-Qual Series 300/500 portable monitors equipped with head sensors (30). Aero-Qual Series 300/500 devices are lightweight, easy-to-use pollutant detectors for determining pollutant concentrations in indoor and outdoor air quality, construction dust, transportation emissions, smog, community exposure studies, and air quality model validation. The operating temperature and relative humidity ranges of the Aero-Qual Series 300 monitor for PM sensor are 0–40°C and 0–90%, respectively. The operating temperature and relative humidity ranges of the Aero-Qual Series 300 monitor for the NO_2_ sensor are 0–40°C and 15–90%, respectively. The PM and NO_2_ sensor heads can measure pollutant concentrations in a range of 0.001–1,000 mg/m^3^ and 0.005–1 ppm, respectively. The device provides immediate, minimum, maximum, and average values depending on the setting, and the PM sensor head measures two values simultaneously namely PM_2.5_ and PM_10_ (22, 32). The Aero-Qual Series 300/500 was set up 2 m above the ground in the middle of the roads in the direction of the pollution source (22). A field observation checklist adapted from previous literature was used to collect data. The monitors were set to record the concentrations at 3-min intervals for 1 h, and the average values were entered into the recording data sheet every 3 min (20, 22). The Aero-Qual Series also has temperature and relative humidity sensors attached to the monitor and at the same time those metrological data were monitored simultaneously with air quality data. The recording data sheet contained the name of the sampling site (identified by an ID for ethical reasons), the date of sampling, and the time of sampling.

### Data quality control

Data quality was ensured by the careful use of the monitoring devices, compliance with the manufacturer’s guidelines, and the use of trained field technicians. The minimum detection limit of the PM sensor is 1 μg/m^3^ with ranges of 1–1,000 μg/m^3^. The minimum detection limit of the NO_2_ sensor head was 0.005 mg/m^3^. The device was supplied with factory calibration with an annual warranty, and the sensor head had different cross-interferences at different concentrations (32). To assure the quality of data, the sampling sites were in an industry-free zone and ensured the absence of any cooking and smoking activities that might bias the concentrations of PM and NO_2_ coming from vehicular sources (33). Data collectors were required to have a master’s degree in Public Health with expertise in Environmental Health and complete a comprehensive two-day training program that covered the introduction, manufacturer’s guidelines, protocols, and mechanisms of operating the Aero-Qual Series 300/500 portable devices attached with PM and NO_2_ sensors heads.

### Data processing and analysis

The data were entered into EpiData (version 3.1) and analyzed using the Statistical Package for the Social Sciences (SPSS) version 26. Descriptive statistics, including minimum, maximum, and mean values, were used to summarize the data. After performing normality and log-normality tests, a non-parametric test was performed. Mann–Whitney U test was used to compare traffic air pollutant between roads with heavy and low traffic volumes. Kruskal-Wallis H tests were used to determine traffic air pollutant between all road types; Kendall’s Tau-b correlation coefficient analysis was performed to assess the correlation between traffic air pollutants and key influencing Metrological Parameters. Finally, stepwise multiple linear regression analysis was employed to examine the relationship between Metrological Parameters and traffic air pollutants.

## Results

### Metrological data

The mean ambient temperature of all road types was 25.5 ± 0.6°C, whereas, similarly, the mean relative humidity was 57.1 ± 3.8% (Table 1).

**Table 1:** Mean values (standard deviation) of meteorological parameters.

Road types	Temperature (°C)	Relative humidity (%)
Asphalt	25.6 (0.7)	57.9 (4.1)
Traffic light	25.7 (0.7)	59.8 (1.8)
Dry weather	25.1 (0.4)	51.9 (3.1)
Red-ash	25.3 (0.8)	57.6 (2.2)
Gravel	25.6 (0.3)	54.2 (2.5)
Cobblestone	25.7 (0.3)	56.1 (3.5)

### Concentration of traffic air pollutants

The mean concentration of PM_2.5_ on heavy-and low-traffic flow roads was 161.6 ± 26.1 μg/m^3^ and 95.9 ± 14.9 μg/m^3^, respectively, while the concentration of PM_10_ on heavy-traffic flow roads was 178.7 ± 20.3 μg/m^3^. Additionally, the mean concentration of NO_2_ on heavy-traffic flow roads was 86.4 ± 14.4 μg/m^3^ (Table 2).

**Table 2:** Mean concentrations of traffic air pollutants between heavy- and low-traffic flow roads in Hawassa City, Ethiopia, 2023.

TRAPs	Heavy-traffic flow roads			Low-traffic flow roads		
	Min	Max	Mean ± SD	Min	Max	Mean ± SD
PM_2.5_ (μg/m^3^)	110	190	161.6 ± 26.1	60	110	95.9 ± 14.9
PM_10_ (μg/m^3^)	150	220	178.7 ± 20.3	70	130	102.3 ± 17.6
NO_2_ (μg/m^3^)	60	120	86.4 ± 14.4	40	90	61.7 ± 14.2

Spatially, the mean concentrations of PM_2.5_, PM_10_, and NO_2_ calculated for the entire study road types were ranging between 83.1 ± 5–164.9 ± 2 μg/m^3^, 91.8 ± 8–185.6 ± 2 μg/m^3^, and 101.9 ± 33.8–172.2 ± 33.8 μg/m^3^, respectively. Additionally, the overall mean concentrations of PM_10_ on asphalt, traffic lights, dry weather, red ash, gravel, and cobble roads were 167.2 ± 23 μg/m^3^, 185.6 ± 20 μg/m^3^, 91.8 ± 8 μg/m^3^, 113.0 ± 11 μg/m^3^, 109.8 ± 23 μg/m^3^, and 94.8 ± 22 μg/m^3^, respectively. In the same manner, the concentrations of NO_2_ on asphalt, traffic light, dry weather, red ash, gravel, and cobble roads were 155.9 ± 20.7 μg/m^3^, 172.2 ± 33.8 μg/m^3^, 108.7 ± 28.2 μg/m^3^, 101.9 ± 33.8 μg/m^3^, 127.8 ± 35.7 μg/m^3^ and 125.6 ± 5.6 μg/m^3^, respectively (Tables 3, 4).

**Table 3:** Spatial variations of traffic air pollutants by road type in Hawassa City, Ethiopia, 2023.

Study road types	PM_2.5_ (μg/m^3^)			PM_10_ (μg/m^3^)			NO_2_ (μg/m^3^)		
	Min	Max	Mean ± SD	Min	Max	Mean ± SD	Min	Max	Mean ± SD
Main asphalt	110	190	155.5 ± 8	140	210	167.2 ± 23	112.8	188	155.9 ± 20.7
Traffic light	140	190	164.9 ± 17	170	220	185.6 ± 20	150.4	225.6	172.2 ± 33.8
Dry weather	60	90	83.1 ± 5	80	100	91.8 ± 8	75.2	131.6	108.7 ± 28.2
Red ash	100	110	107.9 ± 4	100	120	113.0 ± 11	75.2	131.6	101.9 ± 33.8
Gravel	100	110	106.3 ± 2	80	130	109.8 ± 23	94	169.2	127.8 ± 35.7
Cobble	60	100	86.2 ± 21	70	110	94.8 ± 22	112.8	131.6	125.6 ± 5.6
Mean ± SD			129.8 ± 38			140.8 ± 42			140.8 ± 35.7

**Table 4:** Mean concentrations of traffic air pollutants at different monitoring times and locations in Hawassa City, Ethiopia, 2023.

Sampling road types	PM_2.5_ (μg/m^3^)			PM_10_ (μg/m^3^)			NO_2_ (μg/m^3^)		
	15 min	30 min	1-h	15 min	30 min	1-h	15 min	30 min	1-h
Traffic light	150	**150**	150	240	**200**	210	169.2	188	169.2
Traffic light	140	**150**	190	200	**190**	180	169.2	188	188
Traffic light	150	**150**	150	160	**170**	170	206.8	206.8	188
Traffic light	160	**160**	170	170	**160**	180	150.4	169.2	150.4
Traffic light	180	**170**	170	180	**190**	190	169.2	169.2	150.4
Main asphalt	120	**120**	110	180	**180**	160	131.6	131.6	112.8
Main asphalt	200	**200**	190	210	**230**	220	300.8	225.6	**225.6**
Main asphalt	150	**150**	140	170	**160**	170	150.4	150.4	150.4
Main asphalt	220	**200**	190	180	**170**	170	150.4	150.4	150.4
Main asphalt	160	**150**	150	170	**180**	170	112.8	131.6	131.6
Main asphalt	190	**190**	190	220	**200**	180	150.4	169.2	169.2
Main asphalt	130	**130**	130	170	150	150	188	169.2	169.2
Cobblestone	50	**60**	60	70	70	70	131.6	131.6	112.8
Cobblestone	80	**90**	100	100	100	100	131.6	131.6	131.6
Cobblestone	100	**100**	100	110	110	110	169.2	150.4	131.6
Gravel	110	**100**	110	110	90	80	131.6	112.8	112.8
Gravel	120	**110**	110	130	120	120	112.8	112.8	112.8
Gravel	110	**110**	110	110	120	130	112.8	94	94
Red ash	110	**110**	100	120	120	120	169.2	169.2	56.4
Red ash	120	**110**	110	120	120	110	150.4	150.4	131.6
Red ash	110	**100**	100	110	110	100	112.8	75.2	75.2
Dry weather	80	**80**	80	90	90	80	75.2	75.2	75.2
Dry weather	90	**100**	90	100	100	90	112.8	169.2	131.6
Dry weather	90	**90**	90	100	110	100	131.6	131.6	112.8
Mean	132	**131**	130	147	145	141	154.9	149.8	140.8

Bolded numbers indicate; PM_2.5_ > US EPA for 30-min guideline, PM_10_ > US EPA guideline for 30-min, NO_2_ > WHO & Ethiopia for 1-h guideline.

Temporally, the mean concentration of PM_2.5_ during 15-min, 30-min, and 1-h monitoring ranged between 50–220 μg/m^3^, 60–200 μg/m^3^, and 60–190 μg/m^3^, respectively. Likewise, the mean concentration of PM_10_ for 15-min, 30-min, and 1-h measurements ranged between 70–200 μg/m^3^, 70–230 μg/m^3^ and 70–220 μg/m^3^, respectively. Furthermore, the concentrations of NO_2_ during 15-min, 30-min, and 1-h ranged from 75.2–300.8 μg/m^3^, 75.2–225.6 μg/m^3^, and 56.4–225.6 μg/m^3^, respectively. The bolded number indicated that the concentration of traffic air pollutants was high compared to the guideline limits of the US EPA, WHO, and Ethiopian (See footnotes of Table 4).

**Figure 1:**
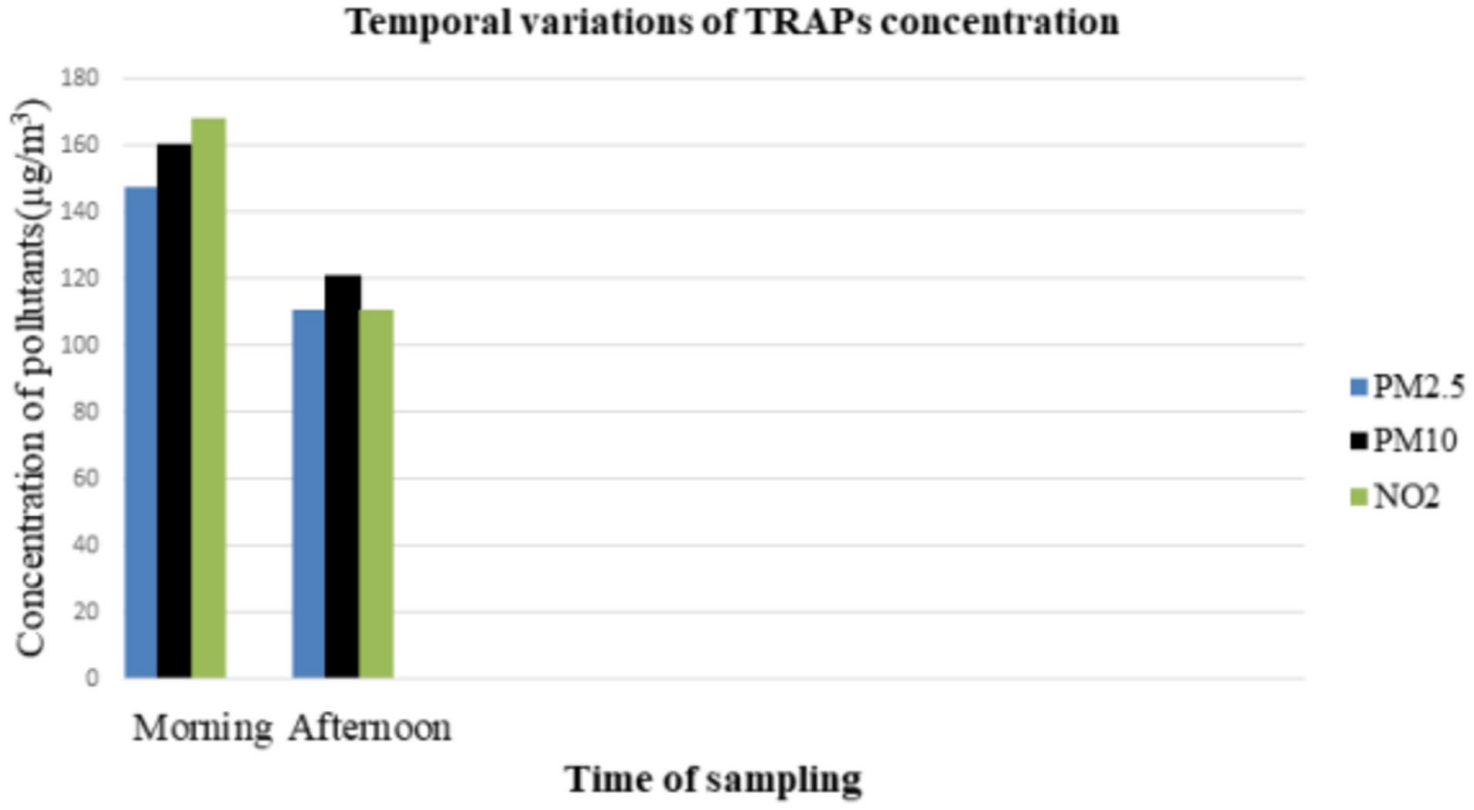
Temporal variations of pollutant concentrations in the afternoon and morning.

Furthermore, the overall mean pollutant concentrations (PM_2.5_, PM_10_, and NO_2_) greatly varied during the morning and afternoon time. The overall mean concentration of PM_2.5_ was 147.4 μg/m^3^ in the morning and 110.1 μg/m^3^ in the afternoon. The mean concentrations of PM_10_ and NO_2_ were 160.1 μg/m^3^ and 167.7 μg/m^3^ in the morning and 120.9 μg/m^3^ and 110.7 μg/m^3^ in the afternoon, respectively (Figure 1, Table 3).

### Comparison of air pollutants on high- low-traffic flow roads

The Mann–Whitney U test was performed to compare pollutant concentrations between heavy and low-traffic volume areas; while the Kruskal-Wallis H test was performed to compare pollutants between asphalt, traffic lights, red ash, cobble, gravel, and dry weather roads. Accordingly, a highly significant difference was observed between heavy-low-traffic flow roads in terms of the concentrations of NO_2_ (Z = −3.406, *p* = 0.001), PM_2.5_ (Z = −4.099, *p* = 0.000), and PM_10_ (Z = −4.157, p = 0.000) at 95% CI, *p* < 0.05. The Kruskal-Wallis H test indicated that there was a significant difference between all road types in terms of NO_2_(χ^2^ = 17.91, DF = 5, *p* = 0.003), PM_2.5_(χ^2^ = 12.91, DF = 5, *p* = 0.003), and PM_10_ (χ^2^ = 24.00, DF = 5, *p* = 0.000) (Table 5).

**Table 5:** Traffic air pollutants concentrations between heavy and low traffic volume areas, in Hawassa City, Ethiopia, 2023.

Variables	Z-value	*p*-value	χ^2^ test	Df	*p*-value
PM_10_ (μg/m^3^)	−4.157	0.000	24.00	5	0.000
PM_2.5_ (μg/m^3^)	−4.099	0.000	12.91	5	0.003
NO_2_ (μg/m^3^)	−3.406	0.001	17.91	5	0.003

### Influence of metrological factors

Changes in the meteorological conditions caused variations in air pollutant concentrations, notably, relative humidity, mean temperature and morning temperature were positively correlated with the concentration of air pollutants, whereas afternoon temperature and humidity were negatively correlated with PM_2.5_ and NO_2_ except for the concentration of PM_10_ (Table 6).

**Table 6:** Kendall’s tau-b correlation coefficient values between air pollutants and metrological parameters in Hawassa City, Ethiopia, 2023.

Variables	PM_2.5_	PM_10_	NO_2_	Temperature (T°C)	Relative Humidity (RH %)
PM_2.5_	1			0.24^a^	0.41^a^
PM_10_		1		0.25^a^	0.48^a^
NO_2_			1	0.21^a^	0.24^a^
T (°C)				1	0.07^a^
RH (%)					1
Morning					
PM_2.5_	1	0.76	0.62	0.37^a^	0.27^a^
PM_10_		1	0.58	0.36^a^	0.27^a^
NO_2_			1	0.33^a^	0.15^a^
T (°C)				1	0.40
RH (%)					1
Afternoon					
PM_2.5_	1	0.70	0.44	−0.06^a^	−0.39^a^
PM_10_		1	0.38	−0.16^a^	0.48^a^
NO_2_			1	−0.02^a^	−0.17^a^
T (°C)				1	0.22
RH (%)					1

a Correlation is significant at the 0.05 level.

To examine the relationship between traffic air pollutant concentrations and meteorological parameters (temperature and relative humidity), Stepwise multiple linear regression (MLR) analysis was performed, and the results are indicated in Table 7. The regression coefficient indicated that there was a direct linkage between traffic air pollutants (as dependent variables) and meteorological factors (as independent variables).

**Table 7:** Multiple regression analysis results for traffic air pollutant concentrations and meteorological parameters.

Parameters	Regression coefficient		
	PM_2.5_	PM_10_	NO_2_
Temperature	0.304	0.208	0.330
Relative humidity	0.455	0.596	0.293

## Discussion

In this study, the mean concentrations of PM_2.5_ (161.6 μg/m^3^) and PM_10_ (178.7 μg/m^3^) on roads with heavy traffic volumes were higher than those of PM_2.5_ (95 μg/m^3^) and PM_10_ (102.3 μg/m^3^) on roads with low traffic volumes. The results of this study were consistent with those of similar studies conducted in Germany (34) and Hungary (35). The PM_2.5_ concentration on roads with high traffic volumes ranged between 110 and 190 μg/m^3^ and that on roads with low traffic volumes was between 60 and 110 μg/m^3^. In addition, the PM_10_ concentration was 150–220 μg/m^3^ on high-traffic roads and 70–130 μg/m^3^ on low-traffic roads. The results of the current study were lower than those of the study conducted in Addis Ababa (36). The reason for this difference could be that the number of vehicles and traffic flows is higher in Addis Ababa than in Hawassa. The mean 30-min concentrations of PM_2.5_ and PM_10_ were between 60–200 μg/m^3^ and 70–230 μg/m^3^, respectively, whereas the mean 15-min concentrations of PM_2.5_ and PM_10_ were between 50–220 μg/m^3^ and 70–240 μg/m^3^, respectively. The results of this study are lower than those of a previous study conducted in Addis Ababa (23). The difference in pollutant concentration could be due to the higher traffic volumes in Addis Ababa than in Hawassa.

The proportion of PM_2.5_/PM_10_ provides important extra information for the air pollution status. Previous studies have found that the PM_2.5_/PM_10_ ratios can provide a series of information such as the cause of air pollution, the air pollution process, and its impact on life and health (37, 38). The ratio of PM_2.5_/PM_10_ in the current study was 0.924. The lower PM_2.5_/PM_10_ ratio indicates coarse particles are dominant and they are more attributed to natural sources (39–41), on the other hand, the higher the ratio of PM_2.5_/PM_10_, the pollution more comes from anthropogenic activities (42–44). The ratio of PM_2.5_/PM_10_ in the previous studies was 0.62 in Wuhan, 0.54, and 0.44 in Beijing in winter and spring, respectively (38, 45). The reason for the higher ratio of PM_2.5_/PM_10_ in the current study could be because the air pollution sources largely come from road traffic air pollution due to high traffic flows. Because, Africa, particularly countries like Ethiopia is the home to second-hand vehicles and poorly maintained old cars, and contributes a large portion of air pollution (15).

In the current study, the mean NO_2_ concentration was higher on roads with heavy traffic volumes (86.4 ± 14.4 μg/m^3^) than on roads with low traffic volumes (61.7 ± 14.2 μg/m^3^). The results of this study were consistent with studies conducted in Italy (46), Morocco (47), and Dire Dawa, Ethiopia (48), which stated that the NO_2_ concentrations were higher on roads with high traffic volumes than on roads with low traffic volumes. The 15-min, 30-min and 1-h mean NO_2_ concentrations in this study were 150.6 μg/m^3^, 148.1 μg/m^3^, and 139.3 μg/m^3^, respectively. The results of this study were higher than those of a similar study conducted in Vietnam (49). The discrepancy in traffic air pollutant concentration could be because many used vehicles are on the road in Africa, and Africa is the home to second-hand vehicles and poorly maintained old cars and these vehicles release more NO_2_ than new vehicles (13).

The current study confirms that there was a highly significant difference between high and low-traffic roads in terms of concentrations of NO_2_ (z = −3.406, *p* = 0.001), PM_2.5_ (z = −4.099, *p* = 0.000), and PM_10_ (z = −4.157, *p* = 0.000) with (95% CI, *p* < 0.05). In this study, the mean concentration of NO_2_, PM_2.5_, and PM_10_ was higher on high-traffic light roads than on paved roads, followed by low-traffic roads. The mean PM_2.5_ concentration on paved roads was 155.5 ± 8 μg/m^3^, on traffic light roads 164.9 ± 17 μg/m^3^, and on low-traffic roads 95 ± 14.2 μg/m^3^. The mean PM_10_ concentration was 167.2 ± 23 μg/m^3^ on asphalt, 185.6 ± 20 μg/m^3^ at traffic lights roads, and 102.3 ± 17.6 μg/m^3^ on roads with low traffic volumes. The NO_2_ concentration was 155.9 ± 0.1 μg/m^3^ on asphalt, 173 ± 0.1 μg/m^3^ at traffic lights roads, and 61.7 14.2 μg/m^3^ on roads with low traffic volumes. The results of this study were in agreement with the study conducted in the USA (18), a meta-analysis from Thailand (50), Norway (19), Uganda (5), and a local study from Addis Ababa, Ethiopia (51), stated that the concentration of traffic air pollutants varies depending on the type of road, the presence of traffic lights, and the characteristics of the roads. The reason for this discrepancy is that the vehicles at traffic lights leave at the same time, and thus, traffic flow is more obstructed. During this time, a higher concentration of pollutants is released than during other times. Another reason could be that asphalted main roads are busier than urban side roads because drivers prefer to travel on these roads due to the convenience of these roads, even if there are other types of roads as an alternative.

According to this study, the mean concentration of traffic air pollutants was higher in the morning than in the afternoon. Accordingly, the mean concentration of PM_2.5_ was 147.4 ± 52 μg/m^3^ in the morning and 110.1 ± 31 μg/m^3^ in the afternoon, whereas, the mean concentration of PM_10_ in the morning and afternoon was 160.1 ± 51 μg/m^3^ and 120.9 ± 39 μg/m^3^, respectively. Besides this, the mean NO_2_ concentration in the morning and afternoon was 167.7 ± 45.1 μg/m^3^ and 110.7 ± 30.1 μg/m^3^, respectively. The results of this study were consistent with those of a study conducted in the USA (18), a literature review in Thailand (50), a study in Malaysia (52), a review in Ethiopia (53), and a study conducted in Nigeria (54), stated that the concentration was higher in the morning than afternoon. The reason for this difference between morning and afternoon could be that the air pollutants in road traffic are less effectively dissolved in the morning than in the afternoon due to the influences of metrological parameters such as temperature, and relative humidity, resulting in low turbulence in the atmospheric air.

In general, the changes in the meteorological conditions cause variations in traffic air pollutants concentration than changes in pollutant emissions over time (55). The Kendall’s tau-b correlation in Table 6 indicated that the mean temperature was positively correlated with the concentrations of NO_2_ (r = 0.21), PM_2.5_ (r = 0.24), and PM_10_ (r = 0.25). During this study, the temperature fluctuated between 22.2–25.8°C and 24–26.7°C in the morning and afternoon, respectively. The results of this study were consistent with those of studies in the USA (56), Addis Ababa, and Dire Dawa, Ethiopia (48, 51), stating that the mean temperature was positively linked with the concentrations of traffic air pollutants. The reason for the positive correlation between temperature and pollutant concentration could be that the temperature in the morning has less influence on the pollutant dispersion rate than the temperature in the afternoon. Therefore, pollutants can remain in the environment for a long time. However, the afternoon temperature in the current study was negatively correlated with NO_2_ (r = −0.02), PM_2.5_ (r = −0.06), and PM_10_ (r = −0.16). The negative results found in the current study were in agreement with those of the study conducted in Vietnam (49), Bangladesh (57), and Thailand (58). The reason for the negative correlation between the afternoon temperature, and concentration of traffic air pollutants could be that the temperature is high in the afternoon, which affects the reaction of traffic air pollutants with the acceleration of atmospheric air.

In the current study, relative humidity was positively linked with the concentrations of NO_2_ (r = 0.24), PM_2.5_ (r = 0.41) and PM_10_ (r = 0.48). During this study, the relative humidity ranged between 57.0–68.0% and 42–62% in the morning and afternoon, respectively. The results of this study were the same as a study conducted in the USA (56), Malaysia (52), and Ghana (59). The reason for the positive correlation could be that a humidified environment does not aid the dissolution of pollutants, and these pollutants can remain in the environment for longer. However, in the afternoon, relative humidity was negatively correlated with PM_2.5_ (r = −0.39) and NO_2_ (r = −0.17), which was in agreement with the study conducted in Thailand (58), and Bangladesh (57).

Furthermore, traffic air pollutants were dependent on the combined effects of metrological parameters (60), and stepwise multiple linear regression analysis were employed to determine the key metrological parameters. Accordingly, the regression coefficient suggested that the concentration of traffic air pollutants was positively linked with metrological parameters. The major metrological parameters that influence the concentration of traffic air pollutants include temperature and relative humidity. The results of multiple regression analysis are given in Table 7. Temperature and relative humidity had a Positive relationship with traffic air pollutants, which was in good agreement with the correlation analysis. Relative humidity was positively associated with PM_2.5_ (R^2^ = 0.455), PM_10_ (R^2^ = 0.596), and NO_2_ (R^2^ = 0.293), which was also observed in China (60), Thailand (58), and India (61). Likewise, Temperature was positively linked with PM_2.5_ (R^2^ = 0.304), PM_10_ (R^2^ = 0.208), and NO_2_ (R^2^ = 0.330). The positive relationship between PM_2.5_ and temperature found in the current study was in agreement with the results of the studies conducted in China (60), Thailand (58), and Nigeria (62), whereas, the positive relationship of PM_10_ and NO_2_ with temperature found in this study was in line with the study conducted in China (60) however, the current study is inconsistent with study conducted in India in that temperature was inversely linked with PM_2.5_ and PM_10_ concentrations (61).

## Conclusion

The total mean concentrations of traffic air pollutants in this study were high compared to the guideline values set by the World Health Organization (WHO). Spatially, the concentrations on traffic light roads were high as compared to the concentrations of air pollutants on main paved roads, followed by the concentration on low-traffic roads. Likewise, there was a difference in pollutant concentration across road types. Temporally, the average traffic air pollutant was higher in the morning than in the afternoon. The PM_2.5_/PM_10_ ratio was high and the value of the ratio confirms that the sources of the air pollution largely depend on anthropogenic sources. Metrological parameters such as temperature and relative humidity were positively correlated with traffic air pollutants and stepwise multiple linear regression analysis show a positive relationship between metrological parameters and traffic air pollutants.

### Limitations of the study

The measurement data from real-time instruments are subject to errors and are recommended to be corrected with reference instruments due to interference with other gases. For example, real-time NO_2_ sensors are affected by Ozone interference. The authors did not check interference from other pollutants.

## Data Availability

The original contributions presented in the study are included in the article/supplementary material, further inquiries can be directed to the corresponding author.
